# An Efficient Moving Target Detection Algorithm Based on Sparsity-Aware Spectrum Estimation

**DOI:** 10.3390/s140917055

**Published:** 2014-09-12

**Authors:** Mingwei Shen, Jie Wang, Di Wu, Daiyin Zhu

**Affiliations:** 1 College of Computer and Information Engineering, Hohai University, Nanjing 211100, China; E-Mail: jiewanghhu@yahoo.com; 2 Key Laboratory of Radar Imaging and Microwave Photonics (Nanjing University of Aeronautics and Astronautics), Ministry of Education, Nanjing 210016, China; E-Mails: wudi82@nuaa.edu.cn (D.W.); zhudy@nuaa.edu.cn (D.Z.)

**Keywords:** space-time adaptive processing, sparse reconstruction, clutter suppression

## Abstract

In this paper, an efficient direct data domain space-time adaptive processing (STAP) algorithm for moving targets detection is proposed, which is achieved based on the distinct spectrum features of clutter and target signals in the angle-Doppler domain. To reduce the computational complexity, the high-resolution angle-Doppler spectrum is obtained by finding the sparsest coefficients in the angle domain using the reduced-dimension data within each Doppler bin. Moreover, we will then present a knowledge-aided block-size detection algorithm that can discriminate between the moving targets and the clutter based on the extracted spectrum features. The feasibility and effectiveness of the proposed method are validated through both numerical simulations and raw data processing results.

## 1. Introduction

Space-time adaptive processing (STAP) has been recognized as a well-established technique in airborne radar for improving the detection performance of moving targets in the presence of clutter. In STAP, a key problem is the estimation of the interference covariance matrix. Typically, covariance matrix is adaptively formulated using the neighborhood range bins under the assumption that the training data are homogeneous, *i.e.*, independent and identically distributed (IID). In practice, the assumption of homogeneous data is routinely violated. As a consequence, the misestimation of the heterogeneous samples may degrade the detection performance of STAP, which has attracted a significant amount of research [1–5].

Since ground clutter is nonhomogeneous, finding sufficient IID secondary data for the detection processing poses the most serious challenge to successfully implementing STAP algorithms. An alternative approach for the detection problem in a heterogeneous environment where training data is severely limited or altogether unavailable is the deterministic STAP approach [6–11], which can remove all undesired contributions from every single range gate, and hence bypass the problem of the required homogeneous secondary data support. The well-know direct data domain (D^3^) methods [6,7] and the maximum likelihood estimation detector (MLED) algorithms [8–11] can only operate with the data under test, so that the performance is not impacted by nonstationarity. Furthermore, a hybrid detector that combines the single-data set (SDS) and two-data set (TDS) algorithms was proposed in [12]. By taking the degree of heterogeneity into account, this approach can outperform both the SDS and TDS algorithms. In this paper, a novel D^3^ STAP algorithm is proposed, which exploits the distinct image features of targets and interference in the high-resolution angle-Doppler image, and discriminates between moving targets and interference based targets on the extracted image features. To efficiently obtain the high-resolution angle-Doppler spectrum from each primary cell only, a reduced-dimension sparse reconstruction (RDSR) algorithm is adapted in the case of D^3^ STAP based on applying the sparse reconstruction in a localized processing region in the transform domain. Moreover, a knowledge-aided block-size detection algorithm based on the resulting angle-Doppler image is also investigated for detecting moving targets, which can effectively reduce the probability of false alarms.

The rest of this paper is organized as follows. In Section 2, the efficient RDSR approach is introduced for the case of angle-Doppler spectrum estimation. Section 3 is devoted to the knowledge-aided (KA) two-stage hybrid detector based on the extracted image features of targets and interference in the angle-Doppler domain. In Section 4, the effectiveness of our approach is tested with both simulated data and measured ground moving target indication (GMTI) data with three-aperture airborne radar. Finally, the conclusions are drawn in Section 5.

## 2. Angle-Doppler Spectrum Estimation Using RDSR

The STAP system under consideration is a pulse Doppler radar residing on an airborne platform. The radar antenna is a uniform linear array, which consists of *N* elements and with the element spacing being half of the wavelength. The platform is at the altitude of *H* and moves with a constant velocity *V*.

**Figure 1.**
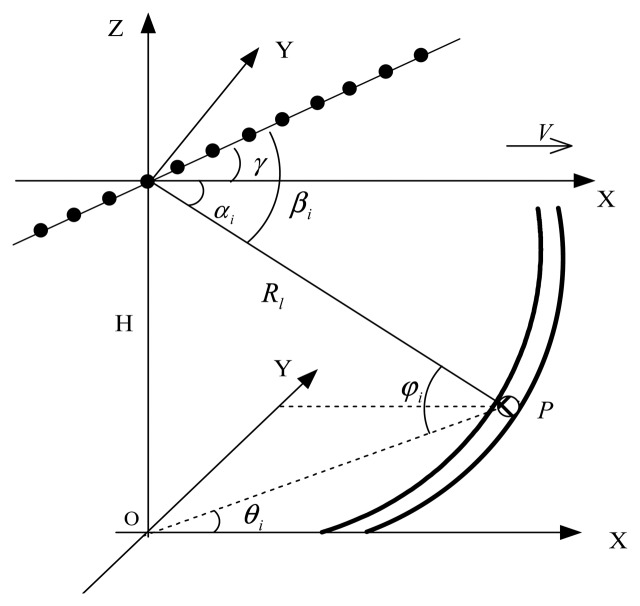
Geometry of non- side looking airborne radar (NSLAR).

Without loss of generality, the non-side looking airborne radar (NSLAR) geometry is shown in Figure 1. The coordinate system assumes that the x-axis is aligned with the flight direction and that the crab angle between the array and the flight direction is *γ*. Suppose that the radar transmits *K* pulses during the coherent processing interval (CPI). Thus, the received clutter data for each range gate *l* can be organized into a space-time snapshot *X_I_* ∈ *C^NK^*^×1^, which can be expressed as

(1) $$X_{l}=\sum_{i=1}^{N_{c}}\sigma_{i}S_{i}+N_{l}$$

where we assume each iso-range to consists of *N_c_* independent clutter patches, whereby *S_i_* indicates the normalized space-time steering vector corresponding to the *i*-th patch, σ*_i_* describes the complex gain, which is proportional to the square-root of the clutter patch radar cross section (RCS), and *N_l_* represents the received noise. For instance, the space-time steering vector corresponding to the *i*-th patch can be written as the Kronecker product of the spatial and temporal vectors:

(2) $$S_{i}=S_{si}⊗S_{di}$$

where *S_si_* =[1 exp(*j*2π*f_si_*)⋯exp(*j*2π(*N*−1)*f_si_*]*^T^*, *S_di_* = [1 exp(*j*2π*f_di_*)⋯exp(*j*2π(*K*−1)*f_di_*)]*_T_* , and the variable *f_si_* and *f_di_* represent the spatial and normalized Doppler frequency, respectively.

**Figure 2.**
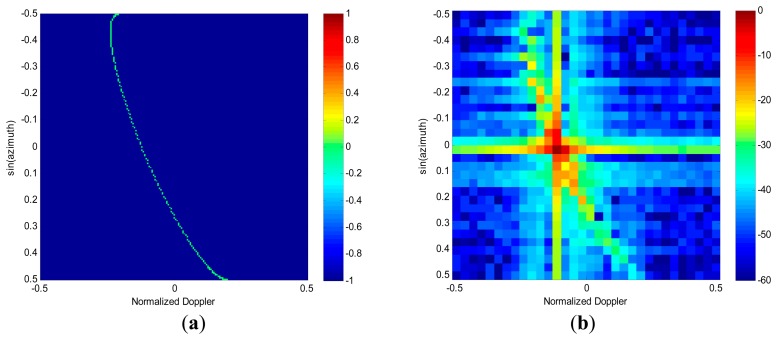
Angle-Doppler spectrum image of clutter. (**a**) Clutter trace. (**b**) 2D fast Fourier transform (FFT).

It is well known that the angle-Doppler spectrum formed by two-dimensional (2D) Fourier transformation suffers from a broad main beam and high side-lobe leakage problem. For instance, given *N* = 32 and *K* =32, *γ* = 30°, with the detailed radar simulation parameters being listed in Table 1. Figure 2a shows the angle-Doppler trace of the received clutter at the range of 30 km. However, the smearing and leakage problems of 2D fast Fourier transform (FFT) are obvious from Figure 2b. As a result, the moving targets may be embedded in the heavy clutter. To solve this problem, some approaches of high-resolution angle-Doppler imaging are considered in [13,14], which have a far superior resolution to the 2D FFT. However, these methods estimate the angle-Doppler spectrum using the full-dimension data, thus requiring large computation times. For instance, the high-resolution angle-Doppler spectrum of each range bin can be estimated via recently proposed sparse reconstruction technique as [14]

(3) $$\begin{array}{l} \hat{\sigma }=\arg\min{\left\|\sigma \right\|}_{0}\\ \text{s}.\text{t}.{\left\|X_{l}-\psi \sigma \right\|}_{2}\leq ɛ \end{array}$$

where ‖·‖*_P_* stands for *L_p_* norm, ψ represents the over-complete basis composed of all desired steering vectors, *σ ^* represents the estimated clutter and targets responses in the angle-Doppler plane, and ε is the error allowance. Equation (3) can be solved via convex optimization. However, since ψ is over-complete, the implementation of sparse reconstruction in the angle-Doppler domain requires multiplications far more complex than *O*[(*NK*)^3^], which makes it impractical.

To reduce both computational burden and nominal training support, several alternate STAP formulations are proposed, which can circumvent joint-domain optimal STAP limitations by applying a cascade or “factored” processing with either the beamformer-Doppler configuration or the opposite configuration. As discussed in [1–3,15], this localized processing idea can be applied with variety of adaptive algorithms such as the high-resolution angle-Doppler spectrum estimation. Intuitively, the aforementioned 2D SR can also be applied separately to both special and temporal parts, together with various processing algorithms for each part. Since in STAP processing, there is a larger number of temporal degrees of freedom (DOF) than spatial DOF, *i.e.*, *K* ≫ *N*, therefore, the introduced RDSR can extract the clutter and target information only in the angle domain within each Doppler bin via post-Doppler transformation. Thus, the RDSR procedure is detailed as follows.

### 2.1. Post-Doppler Transformation

For notational convenience, the received data *X_l_* can be reshaped into a *N* × *K* matrix as

(4) $$X_{l}^{\prime }={\left[S_{l\_1}\;S_{l\_2}\cdots S_{l\_K}\right]}_{N\times K}$$

where *S_l_*___*_i_* represents the spatial snapshot vector from the *N*-element array for the *i*-th pulse of a *K*-pulse CPI. Therefore, the data in the space-time domain can be first transformed to the element-Doppler domain via one-dimensional Fourier Transform, which can be implemented as

(5) $$D_{\_}X_{l}=X_{l}^{'}F_{D}^{H}=[S_{Dl\_1}\;S_{Dl\_2}\cdots S_{Dl\_K}]$$

where *F_D_* comprises the Fourier Transform weights, and *S_Di_*___*_i_* represents the element-Doppler vector corresponding to the *i* th Doppler cell. It should be noted that a weight function such as Chebyshev window could be applied prior to the transformation to limit the sidelobe level in the Doppler domain.

As shown in Figure 2, the clutter within each Doppler bin is significantly sparser in the angle domain. Therefore, due to the post-Doppler transformation, the spectral distribution of each independent scatterer now can be obtained through SR only in the angle domain, which results in the advantage of computational efficiency than the direct implementation of SR in angle-Doppler domain [14]. In addition, with respect to the direct implementation of SR, this post-Doppler transformation also improves both signal-to-noise ratio (SNR) and clutter-to-noise ratio (CNR), which consequently increases the accuracy of angle-Doppler spectrum estimation [15].

### 2.2. Spatial SR

For each Doppler bin we can scan over the angle dimension to form the two-dimensional spectral distribution for moving targets as well as the clutter-and-noise using the element-Doppler data. The element-Doppler data for the *i*-th Doppler bin can be expressed as

(6) $$S_{Dl\_i}=\sum_{j=1}^{N_{i}}K\sigma_{i\_j}S_{si\_j}+N_{j}$$

where *σ_i_*___*_j_* and *S_si_*___*_j_* denote the complex amplitude and spatial steering vector corresponding to the *j*-th independent scatterer located in this Doppler cell, respectively, *N_j_* represents the noise, and *K* is the gain due to the coherent accumulation of the Fourier Transform in the time domain.

From the above discussion, it is clear that both the targets and clutter responses in the angle domain can be computed, similarly to Equation (3), as follows [14–16]

(7) $$\begin{array}{l} {\overset{⌢}{A}}_{i}=\arg\min{\left\|A_{i}\right\|}_{1}\\ s.t.{\left\|S_{Dl\_i}-\psi_{i}A_{i}\right\|}_{2}\leq {ɛ}_{i} \end{array}$$

where *Ȃ_i_* is the estimated spatial distribution of targets and clutter within the *i*-th Doppler cell, ψ*_i_* is an over-complete representation in terms of all possible source locations, and ε*_i_* is the parameter specifying how much noise we wish to allow. We assume that the angle grids for both SR and RDSR implementations are given as the same number as *N_s_* = ρ*_s_N*, and that ρ*_s_* is set to be much greater than the one to achieve the super-resolution source location. However, the parameter of ρ*_s_* should be selected with further consideration of radar operating parameters, such as numbers of coherent pulses and antenna array elements. In our simulation, the value of ρ*_s_* is selected to be ρ*_s_*=3.

Applying this procedure repeatedly for all Doppler cells, the high-resolution 2D spectral distribution of both moving targets and clutter can be obtained. The total complex multiplications for the RDSR implementation is *O*[*KNsN*^2^]. Therefore, in contrast to 2DSR, it is clear that the proposed RDSR is much more efficient.

## 3. KA Image Feature-Based Moving Targets Detection

**Figure 3.**
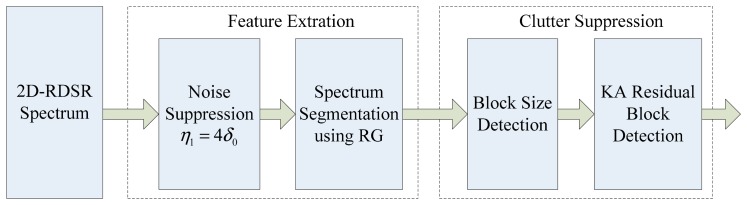
Flowchart of knowledge-aided (KA) image feature-based moving targets detection.

In practice, it is highly desirable to automatically detect moving targets from interferences in the angle-Doppler plane. However, as we will see later in Section 4, the targets and clutter are clearly separated in the angle-Doppler domain due to the high resolution of the RDSR approach, which is very dependable, and the overall detection performance, which are basically determined by the processing of a 2D spectrum estimation. In [17], an image feature-based space-time processing (IFSTP) algorithm is developed, which extracts targets and interference features in the angle-Doppler domain using the region growing approach, and an innovative block-size detection algorithm is proposed to discriminate between the moving targets and interference. However, the straightforward implementation of IFSTP in our study may lead to degraded detection performance, since the clutter distribution obtained via RDSR is not a tilted ridge but composed of individually distinguished scatterers, which may be mis-detected as moving targets and result in an increase of false alarms. To overcome this shortcoming, the prior information of clutter distribution can be used to enhance the detection performance. Hence, a KA image feature-based moving target detection algorithm is presented in this section, and its flowchart of implementation is shown in Figure 3.

### 3.1. Feature Extraction

To remove the white noise, a clamping processing prior to the extraction of clutter and targets should be applied to all pixels of the spectrum image. The threshold value is set to be [18]

(8) $$\eta_{1}=4\delta_{0}$$

where δ_0_ is the estimated standard deviation of the white noise. After the clamping processing, the remaining nonzero pixels are either targets signals or clutter.

Therefore, both the clutter and targets can be extracted using the region growing algorithm [19], and the spectrum image is segmented into pixel blocks consisting of consecutively connected nonzero pixels. For instance, the range *R* between a pixel *q* and a pixel block is defined as the minimum distance between *q* and any pixel in the *i*-th pixel block *B_i_*

(9) $$R=\underset{j}{\min}\left\|p_{j}^{i},q\right\|,1\leq j\leq L$$

where $p_{j}^{i}$ is one of the *L* pixels in *B_i_*, and the distance between the two pixels $p_{j}^{i}$ and *q* is calculated as follows

(10) $$d_{P_{j}}^{q}=\left\|p_{j}^{i},q\right\|=\sqrt{{\left(\frac{R_{a}}{r_{a}}\right)}^{2}{\left(i_{a}-j_{a}\right)}^{2}+{\left(i_{d}-j_{d}\right)}^{2}}$$

where (*i_a_*,*i_d_*) and (*j_a_*,*j_d_*) are the angle-Doppler index grids corresponding to $p_{j}^{i}$ and *q*, respectively. *R_a_* and *r_a_* represent the actual image resolution in angle and the parameter used to adjust the effect of the angle resolution on the defined range. Therefore, if *R* is not larger than the pre-defined distance *D*, the pixel *q* is available for merging into *B_i_*. The detailed description of the region growing method can be found in reference [19].

### 3.2. Moving Target Detection

Due to the feature extraction, the spectrum image becomes a collection of pixel blocks that are either targets or clutter. In [17], a new detection criterion based on block size was introduced to determine a block *B_i_* in which the size *S_i_* in the angle-Doppler domain is a target or interference. The block size *S_i_* of *B_i_* is defined as the maximum distance of any two pixels inside the block [17], *i.e.*,

(11) $$\begin{array}{ll} S_{i}=\underset{j,k}{\max}\left\|p_{j}^{i},p_{k}^{i}\right\|, & 1\leq j,k\leq Z \end{array}$$

where *Z* is the number of pixels in the block *B_i_*. Based on the block size, the reconstructed continuity clutter can be directly determined as

(12) $$S_{k}>\beta_{1}$$

where *β*_1_ is the detection threshold and can be derived from the resolution of the spectrum image. In [17], this detection threshold is suggested to be *β*_1_ =4∼8.

In contrast to 2D FFT, the spectrum image obtained via RDSR may lead to the clutter distributed non-continuity in the angle domain, because of the high resolution and the lack of leakage problem. Therefore, the isolated clutter points will also be mis-detected as moving targets. This, in turn, results in a high probability of false alarm.

Recently, the prior knowledge on operating parameters of the radar can be used to assist and improve the performance of GMTI [20,21]. Therefore, we assume that the instantaneous radar parameter is available, and a knowledge-aided detector can be employed in the second detection stage.

The blocks passing the first detection stage are concentrated and are of a small size, hence the corresponding angle—Doppler information can be obtained

(13) $$\left(\beta_{i},f_{i})=\text{pos}(p_{mid}^{i}\right)$$

where $p_{mid}^{i}$ represents the middle pixel of *B_i_* in the angle-Doppler plane, *β_i_* and *f_i_* are the angle and Doppler axis values, respectively. However, the instantaneous radar parameter can be incorporated into the clutter suppression. As shown by Equation (2), the Doppler frequency corresponding to the clutter scatterer located at angle *β_i_* can be calculated, and its value should be *f̂_i_* [2]. It is clear that when *B_i_* is clutter, its Doppler frequency *f_i_* is approaching *f̂_i_*, *i.e.*, *f_l_* ≈ *f̂_l_*. As a consequence, the moving targets satisfy

(14) $$\left|f_{l}-{\hat{f}}_{l}\right|>\beta_{2}$$

where |·| represents the absolute value operator, and *β*_2_ is the threshold. *β*_2_ is determined by the Doppler resolution of the angle-Doppler spectrum image.

The detection process in both Equations (12) and (14) is repeated for all pixel blocks in the transformed image. Therefore, the residual blocks are declared as the desired moving targets. Note that the target parameters of both angle location and radial velocity can also be obtained via Equation (13).

## 4. Performance Analysis

In this section, we first quantitatively evaluate the proposed scheme using simulated data. Then, the real measured data of three aperture airborne radar for synthetic aperture radar (SAR) GMTI experiments are also processed to verify its robustness and practicability.

### 4.1. Simulated Data

Consider a non-sidelooking airborne early warning radar with *N*=32 antenna elements and *K*=128 pulses in one CPI. The radar system parameters are listed in Table 1.

**Figure 4.**
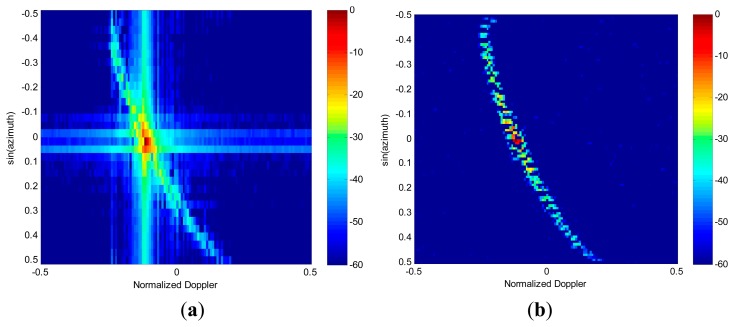
Angle-Doppler spectrum image. (**a**) 2D FFT. (**b**) reduced-dimension sparse reconstruction (RDSR).

Giving the clutter space-time snapshot with range cell *l*=100, Figure 4a shows the angle-Doppler spectrum via the 2D Fourier Transform. As expected, the smearing and leakage problems of FFT are obvious. The RDSR image, see Figure 4b, is obtained using a uniform angular scanning grid with the number of *N_s_*=3*N*. Note that the RDSR image has much better resolution and much lower sidelobe level than that of 2D FFT and that the RDSR estimated clutter spectrum is well focused along the clutter trace.

**Figure 5.**
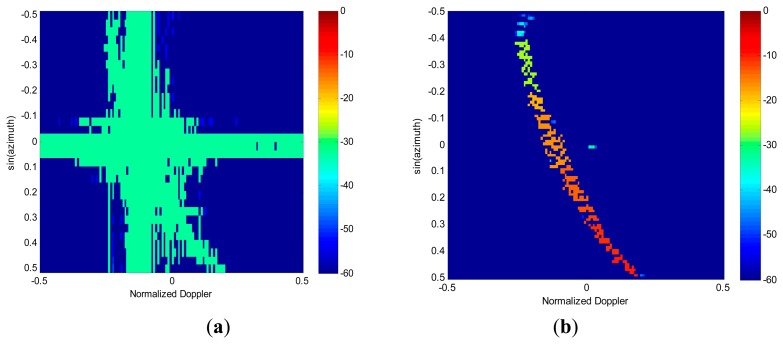
Feature extraction via RG algorithm. (**a**) 2D FFT. (**b**) RDSR.

Next, the performances of spectrum image feature extraction are analyzed in the presence of both clutter and moving targets. We insert a target at the azimuth angle of 0°, which is aligned with the main beam direction. The target is assumed to have a radial velocity of 10 m/s, and its signal-to-clutter-plus-noise ratio (SCNR) is set to be −25 dB. For extraction of targets and interference, the RG algorithm is applied to both images obtained in Figure 4, respectively. The nonzero pixel blocks generated by RG for the case of 2D FFT spectrum and RDSR spectrum are displayed in Figure 5. The position of each block is indicated by its color, as indexed by the corresponding color bar. As shown in Figure 5a, both the clutter and the target are concentrated in the same block due to the smearing and leakage of FFT. Therefore, the moving target is embedded in the clutter. In Figure 5b, it is clear that the target signal and the clutter become clearly visible, but the ridge of clutter is segmented into different groups due to its non-continuity distribution in the angle plane. However, they can easily be eliminated as non-targets in the detection process.

**Figure 6.**
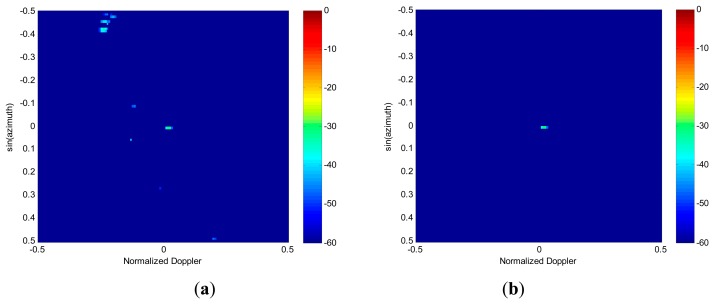
Outputs of Knowledge-aided image feature based detection algorithm. (**a**) Results of the first detection stage. (**b**) Results of the second detection stage.

Finally, the KA image feature-based detection algorithm is carried out for all blocks in Figure 5b. The threshold block size used for target detection in the first detection stage is selected as *β*_1_=5. From Figure 6a, it can be seen that most of the clutter can be efficiently suppressed by block-size detection. Although the isolated clutter blocks that meet the target size criterion are censored, they are passed on to the next stage of detection. The results of the KA detector are shown in Figure 6b. It is evident that with the help of prior knowledge, the residual clutter is well cancelled and the target block is correctly detected. Therefore, the simulation results suggest that the proposed detection algorithm is capable of correctly detecting targets in the angle-Doppler spectrum image estimated via RDSR. Furthermore, the target parameters of location and velocity are also obtained.

### 4.2. Measured Data

Three-channel real data acquired by an X-band SAR-GMTI system is also used to validate the proposed algorithm. System parameters for the SAR-GMTI experiment are shown in Table 2.

**Figure 7.**
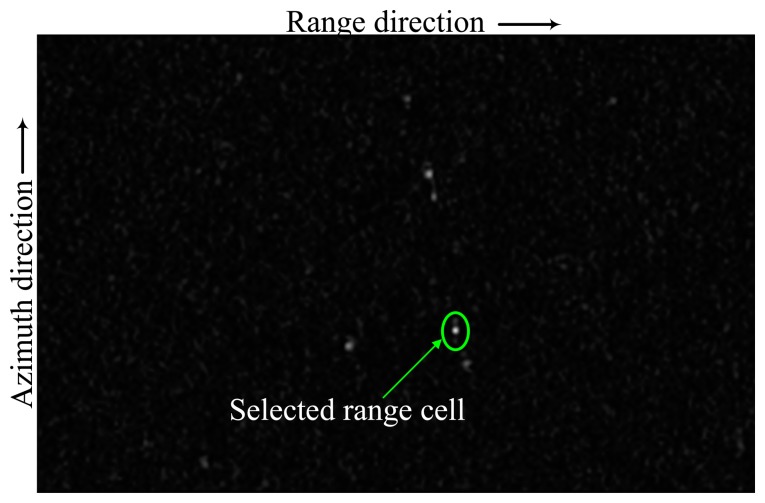
Results of factored space-time adaptive processing (STAP) processing.

We intercept a scene with 200 range cells in one antenna look direction, and there are six moving vehicles within this scene. Figure 7 shows the clutter suppression result via the well-established factored STAP algorithm, in which the six moving targets are visible. Therefore, the snapshot of target range cell *l*=17 is selected for performance assessment.

**Figure 8.**
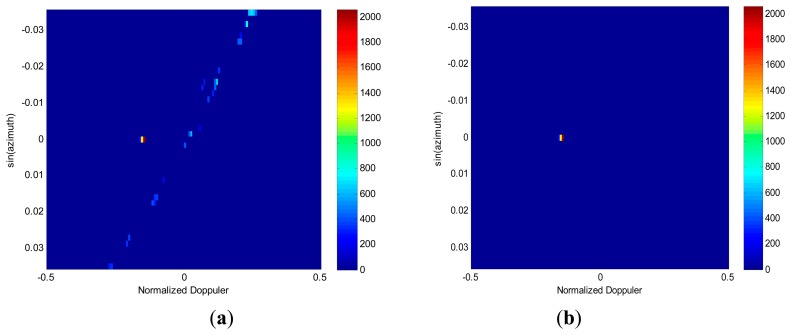
Performance assessment using the three-channel SAR/GMTI real data. (**a**) 2D RDSR spectrum estimation. (**b**) Moving target detection.

Figure 8a gives the spectral estimation in angle-Doppler domain via RDSR where the noise has been suppressed by the clamping processing. Due to the large distance between subapertures, the scanning azimuth angle ranges only from −2° to 2°, and the main beam direction is compensated for by the azimuth angle 0°. It can be seen that both clutter and the moving target are well separated. Figure 8b displays the results after applying the knowledge-aided image feature based detection algorithm. It is clear that only the true target is declared. Thus, the effectiveness of the proposed algorithm is verified by processing the three-channel SAR-GMTI real data.

## 5. Conclusions

This work described an efficient direct data domain STAP scheme for moving target detection in airborne radar systems. Firstly, it employed a low-complexity RDSR approach to estimate the angle-Doppler spectrum for each range cell. Therefore, due to the high resolution and low sidelobe level of the spectrum image formed via RDSR, the moving targets could be effectively detected based on distinguishing image features of targets and interference in the angle-Doppler plane. Processing results with both simulated and measured data have shown that the proposed method can robustly suppress clutter in the presence of non-continuity distribution in the angle domain, and that the true targets can be correctly detected. In addition, by avoiding the requirement of estimating the clutter-and-noise covariance matrix, this algorithm is particularly suitable for applications in highly heterogeneous clutter environments.

## Figures and Tables

**Table 1.** Simulation Parameters for airborne early warning (AEW) radar.

Carrier Frequency	1.35 GHz
Pulse Repetition Frequency (PRF)	5000 Hz
Bandwidth	5 MHz
Array Element Number	32
Platform Velocity	150 m/s
CPI Pulse Number	128
Platform Height	8000 m
Element Spacing and Wavelength Ratio	1/2
Clutter-to-Noise Ratio (CNR)	40 dB

**Table 2.** System Parameters for synthetic aperture radar (SAR)-ground moving target indication (GMTI) experiment.

Carrier Frequency	9.72 GHz
Range Bandwidth	10 MHz
PRF	1250 Hz
Phase center separation	0.35 m
Platform Velocity	110 m/s
Pulse Number	128
Platform Height	5300 m
Swath central range	24 km
